# The Development of Pathways in Palliative Medicine: Definition, Models, Cost and Quality Impact

**DOI:** 10.3390/healthcare7010022

**Published:** 2019-02-01

**Authors:** Laura Finn, Sonia Malhotra

**Affiliations:** 1 Division of Hematology and Bone Marrow Transplant, Department of Internal Medicine, Ochsner Medical Center, 1514 Jefferson Highway, New Orleans, LA 70121, USA; 2 Department of Palliative Medicine & Supportive Care, Section of General Internal Medicine & Geriatrics, University Medical Center, Tulane University School of Medicine, 2000 Canal Street, New Orleans, LA 70112, USA; smalhotr@tulane.edu

**Keywords:** palliative care, palliative medicine, serious illness, cost-effectiveness, hospice, quality, clinical pathways

## Abstract

Palliative Care and its medical subspecialty, known as Palliative Medicine, is the care of anyone with a serious illness. This emerging field includes Hospice and comfort care, however, it is not limited to end-of-life care. Examples of the types of serious illness that Palliative Medicine clinicians care for include and are not limited to hematologic and oncologic diseases, such as cancer, advanced heart and lung diseases (e.g., congestive heart failure and chronic obstructive pulmonary disorder), advanced liver and kidney diseases, and advanced neurologic illnesses (e.g., Alzheimer’s and Parkinson’s disease). In the past decade, there has been tremendous growth of Palliative Medicine programs across the country. As the population of patients with serious illnesses increases, there is growing concentration on quality of care, including symptom management, meeting patients’ goals regarding their medical care and providing various types of support, all of which are provided by Palliative Medicine. In this review article we define Palliative Medicine, describe care pathways and their applicability to Palliative Medicine, identify different models for Palliative Care and provide evidence for its impact on cost and quality of care.

## 1. Introduction

### 1.1. Objective

There is practically a complete absence of literature regarding care pathways in Palliative Medicine, laying bare a wide-open knowledge gap of their potential use and benefits in this field of medicine despite tremendous growth of this specialty across our country. Both an aging population and significant advances in medicine increase the need for better Palliative Medicine utilization in overall healthcare. The objective of this review article is to briefly define Palliative Medicine and its components, to describe the very rare use of care pathways in the field, and further discuss models of Palliative Medicine practice, including the cost and quality of care that should allow for care pathway development. 

### 1.2. Definition of Palliative Medicine

**Figure 1:**
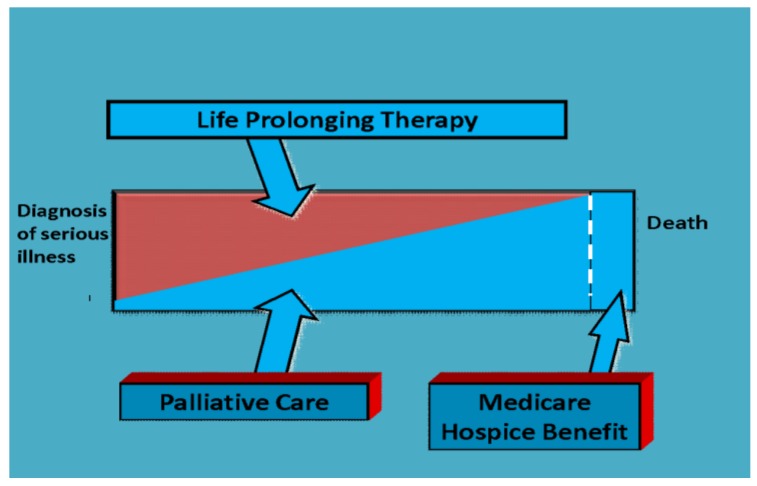
Palliative Medicine’s Role during Illness. Adapted from “the integrated model of care” proposed by the World Health Organization in 1990 [1].

According to the Center to Advance Palliative Care (CAPC), Palliative Care and its medical subspecialty, known as Palliative Medicine, are defined as the “specialized medical care for people living with serious illness.” [1]. Palliative Care is the relief of suffering and soothing of symptoms caused by disease or illness, this is care provided by any member of a healthcare team. Palliative Medicine is a fellowship trained medical subspecialty providing expert level Palliative Care to patients with serious illness. The illness may be considered serious in a variety of ways, including illness that is incurable and/or life-threatening, that manifests with difficult symptoms or requires multiple hospital admissions for treatment, or illness that drastically changes a patient’s quality of life. Palliative Medicine is provided by a workforce of providers, including physicians, advanced practice providers, nurses, chaplains and social workers. Some teams may include art and music therapists, pharmacists and child life therapists as well. These providers work with patients’ specialists to provide pain and symptom management, communication expertise, emotional, spiritual and psychosocial support as well as end-of-life care when appropriate. The goal of Palliative Medicine is to alleviate the burden of serious illness through the improvement of quality of life by addressing gaps in symptom management and communication. Palliative Medicine involvement is appropriate for any age and any stage of a serious illness and can be provided alongside curative treatment with additional services if death occurs (Figure 1).

**Figure 2:**
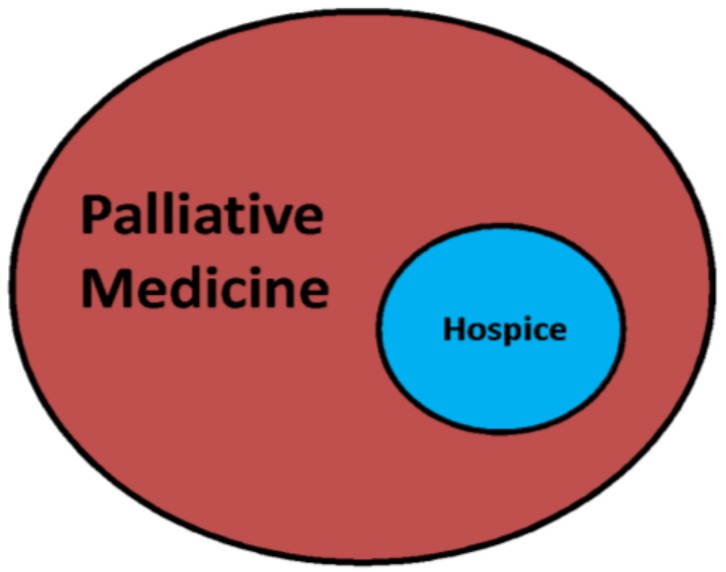
Palliative Medicine and Hospice Care. Adapted from Palliative Medicine and Geriatric Emergency Care [2].

Palliative Medicine and Hospice (end-of-life care) are often thought to be synonymous but Hospice is a small role of Palliative Medicine (Figure 2). End-of-life care is care of a patient in their final hours or days is one aspect of Palliative Medicine, often performed through Hospice care. Hospice care is the treatment of a person at the end of life, which is recommended when a patient’s prognosis is six months or less. Hospice care is available to patients through the Medicare Hospice Benefit covering the cost of end of life care for patients with Medicare Part A and certification of a terminal illness by both a hospice doctor and the patient’s usual physician (Figure 1). Hospice also provides caregiver and family support and bereavement services after a patient’s death. Palliative Medicine teams often coordinate and/or provide hospice care.

### 1.3. Care Pathways and Use in Palliative Medicine

The epidemiology of dying is evolving. The verification of a terminal illness from a patient’s usual physician is important as death now often follows an extended period of health decline without a clear entry point to the dying phase. The use of advanced interventions has made conversations around health care planning and eventual hospice care that much more important. Novel therapies are also disrupting abilities to accurately prognosticate outcomes of severe illness, leading some health systems to use pathways to trigger Palliative Medicine consults, though at present care pathways in Palliative Medicine are rare. The pathways health systems use to trigger Palliative Medicine consults often include the following [3]:

◦ Readmission from long term care facilities
◦ Frequent admissions in a set time (e.g., greater than three admissions in one month)
◦ More than one admission for a terminal illness
◦ Advanced or metastatic cancer, which is not expected to benefit from cancer-directed therapy
◦ Difficult symptom management, which needs specialty intervention
◦ A discordance between goals of care and advanced directives
◦ Critically ill/terminally ill patients without advanced directives

The European Association of Care Pathways consensus meeting in 2005 defined a care pathway as “a methodology for the mutual decision making of care for a well-defined group of patients during a well-defined period”. Defining characteristics of care pathways include an explicit statement of goals and key elements of care based on evidence, best practice and patients’ expectations. The definition may also include facilitation of how communication occurs, coordination of roles and sequencing of the activities of the multidisciplinary care team between patients and their relatives. The documentation, monitoring and evaluation of variances and outcomes as well as the identification of appropriate resources must also occur in care pathways [4].

Primary clinical care pathways are structured—sometimes multidisciplinary—plans used by health services to outline algorithms of care for patients with specific diagnoses or clinical problems. These pathways have developed as treatment decisions, which evolved from evidence-based medicine. The pathways provide a link to create local protocols for clinical practice from guidelines [5]. Often primary clinical care pathways provide a standardized approach towards a specific clinical problem and impact hospital metrics, including the length of stay, cost and quality of care, leading to the ability to measure outcomes as a result of applying these pathways.

A non-small cell lung cancer (NSCLC) pathway has been used at Dana–Farber Hospital in Boston since 2014. The development of this pathway occurred due to a large number of patients being seen with NSCLC, the complexity of this cancer and wide variation in oncologists’ practices in treating this cancer. A standard algorithm of treatment options was created with flags requiring override that pop-up in the electronic medical records when clinicians order therapies that deviate from the algorithm. The Dana–Farber NSCLC pathway has decreased the outpatient cost of care per patient by nearly $15,000 while maintaining median survival. The 25% reduction in cost was attributed widely to the decreased use of antineoplastic drugs as a part of the pathway. Using this care pathway, the institution saw cost savings without compromise of care or survival outcomes. Researchers at Dana–Farber are also evaluating whether the NSCLC pathway has an impact on patient emergency room visits and hospitalizations [6]. As hospitals look to impact the cost and quality of care, hopefully, more care pathways in Palliative Medicine will develop in the future.

The Liverpool Care Pathway was developed in 1997 as a pathway to provide a template to non-Palliative Medicine specialty providers of evidence-based, multidisciplinary care of patients at the end-of-life and included support for caregivers and families with the intent to provide Palliative Care across the entire United Kingdom health system. The data from the program showed that it improved knowledge of when and how to stop futile therapies and how to communicate with patients and families about death and dying. This pathway also provided evidence that improving communication between medical staff and between the medical staff and patients and their families had positive outcomes for patients [7]. The program was abruptly discontinued in 2014 due to various criticisms from national reviews.

The discontinuation of the Liverpool Care Pathway, which was the most used end-of-life care pathway ever, raised the question of whether end-of-life care pathways for the treatment of the dying were effective. In a 2016 Cochrane review of end-of-life care pathways, only one study met inclusion criteria and only 34% of patients in the study were cared for in accordance to the pathway. Minimal patient outcomes were reported. There are limited studies to provide sufficient data regarding the clinical effectiveness of end-of-life care pathways [8].

While few end-of-life care pathways have been used, there are even fewer pathways focused solely on Palliative Medicine for patients earlier in their disease trajectory of serious illness. Evidence supporting care pathways for acute and chronic disease management can be drawn from many areas of medicine and some have Palliative Medicine integrated into them [9]. Prominent examples are the pathways of cancer management published and regularly updated by the National Cancer Care Network (NCCN). Palliative Medicine now has a primary pathway under the NCCN as a guide to Oncologists [10].

The true benefit of a care pathway is applying consistency to the way a medical subspecialty is practiced and the data it can provide, by allowing for comparisons of treatment methods and patterns. Pathways also allow consistency in teaching. However, care pathways also allow for flexibility to make exceptions when clinicians need to use their clinical judgment to solve a problem. Creating care pathways within Palliative Medicine provides the ability to standardize features of the practice, including the structure, funding, composition of team members and services provided. However, there are challenges to creating Palliative Medicine care pathways for chronic, non-malignant diseases, such as advanced neurologic or cardiac diseases. These challenges include the wide variety of diagnoses that fall under these medical specialties, difficulty in predicting duration and course of illness, types of medical interventions required to manage the disease and lack of Palliative Medicine clinician experience with these diseases or their advanced medical interventions [10].

Although the NCCN has established a Primary Palliative Medicine care pathway, there are challenges to this pathway, even in Oncology, including the need for a wide variety of services for these patients. In cancer care, Palliative Medicine clinicians often see patients at diagnosis if symptoms are distressing and follow them through survivorship if long-lasting effects of treatment remain [10]. Another unique challenge in Oncology is that each patient experience is unique and the components of Palliative Medicine required to meet these individual needs are often not available due to lack of funding or resources. Currently, the American Society of Clinical Oncology (ASCO) recommends that all patients with a new diagnosis of cancer have an interdisciplinary Palliative Medicine evaluation within eight weeks of diagnosis, that focuses on symptom management, clarification of treatment goals, supporting of distress and coordination of care [11]. Following this care pathway is dependent of the region of practice and what types of Palliative Medicine services are available.

Integrated care pathways are those that include Palliative Care as a part of usual patient care, with adjustments to their involvement as the illness progresses. Research has shown that improvements occur within the overall quality of care and with Palliative Medicine delivery when integrated care pathways are used. Examples of integrated pathways include the care of patients with advanced heart failure who are generally referred when they develop symptoms of New York Heart Association (NYHA) Class III or IV or American College of Cardiology (ACC) or American Heart Association (AHA) Stage D disease [12,13,14,15,16,17,18]. Patients with heart failure have a large burden of symptoms including dyspnea and fatigue, high psychosocial distress of patients and caregivers and the increasing need for advance care planning as their disease progresses. Additionally, the discussion surrounding complex medical decisions, involving organ transplant and advanced mechanical circulatory options is crucial. One aspect required for evaluation for a left ventricular assist device (LVAD), a type of mechanical circulatory option, is an evaluation by a Palliative Medicine clinician for symptoms and preparedness planning.

Few and far, both primary and integrated care pathways in Palliative Medicine require further research. As of now, two Cochrane system updates did not identify any studies that met minimal criteria for analysis inclusion to support use and dissemination of Palliative Care pathways [19].

## 2. Models of Palliative Medicine Delivery

Palliative Medicine can be provided in almost any care setting including inpatient hospital consults (consultative), dedicated hospital-based Palliative Medicine units, outpatient clinics, assisted living and long-term care facilities and home-based care [1]. Palliative Medicine services may be provided in person, by video or by telehealth services [20]. Palliative Care may also be divided in tiers of care from primary to tertiary Palliative Medicine [21].

### 2.1. Inpatient Palliative Care

The two primary models of inpatient Palliative Medicine are: a) Consultative services and b) inpatient units. Inpatient consult services may include a single clinician, an interdisciplinary team, or a Palliative Medicine hospital unit [22]. Inpatient Palliative Medicine should have the capability to provide consultations from the emergency room to the intensive care unit [23]. Inpatient Palliative Medicine units are found in larger hospitals or health care systems with the ability to admit patients for symptom management, support severely distressed families or to manage the imminently dying patient. 

Consultative Palliative Medicine is the most common form of inpatient practice and is the same as any other medical specialty consult provided upon request of the expert. Physicians who consult Palliative Medicine typically seek support in four domains:

1. Pain and symptom assessment and management
2. The communication between healthcare teams and patients and/or family regarding goals of care and advanced medical decision making
3. The provision of support to patients, families, and health care teams
4. End-of-life care, comfort care and Hospice services

Consultative Palliative Medicine differs from practices with full services of independent outpatient clinics, inpatient units, and admitting service privileges. Across hospital programs of varying sizes in the United States, Palliative Medicine inpatient services have a mean penetration of 4.4%, which has increased by 63% since 2008. The development of Palliative Medicine units has grown tremendously since the early days of strictly inpatient consult services. From 2008 to 2014, consult volumes increased by 91% [24]. In 2011, 65% of hospitals had an inpatient Palliative Medicine service with larger hospitals (>300 beds) representing more than 85% of programs. The factors associated with having inpatient Palliative Medicine services included not-for-profit institutions, institutions that owned a hospice program, status as an American College of Surgery approved cancer hospital and having a higher percentage of patients with a college education [25].

### 2.2. Outpatient Palliative Care

Due to challenges in implementation and the current Palliative Medicine workforce shortage, less than half of the nation’s Palliative Medicine programs have outpatient services [26,27]. Healthcare is moving towards the development of “medical homes” or centers that serve as primary care and subspecialty centers without patients needing to go to multiple locations for care. Research has shown that the integration of outpatient Palliative Medicine into large private oncology systems improves clinician and patient satisfaction and referrals increase rapidly [28]. The goal in outpatient Palliative Medicine is to save the primary team time by addressing symptom burden, goals of care conversations, advance care planning and coordination of care. 

Overall outpatient Palliative Medicine can deliver care earlier during a serious illness. Methods should be established to consider outpatient Palliative Medicine referral patterns, which will help administrators set benchmarks to assess program quality, allow for research of patient concerns and allow development of care pathways [29].

### 2.3. Community-Based Palliative Care

Community-based Palliative Care (CPC) is an emerging model of Palliative Medicine that spans through the inpatient and outpatient settings to provide serious illness care at home or in a long-term care setting. The goal of CPC is to provide longitudinal care across patient transitions and serve as a bridge between Palliative Medicine and Hospice [30]. Database collection and collaboration among regional settings are occurring to understand the types of patients being referred to CPC, symptom profiles and change in symptoms after visits, which may be used to develop specialized care pathways in this unique care setting [31].

## 3. Cost & Quality Impact of Palliative Medicine Models and Pathways 

The data from randomized control trials in nearly all specialties—including heart failure, dementia, and end-stage renal disease —in Palliative Medicine, clearly show that this specialty improves patient quality of life, quality of care and reduces the cost of care [32,33,34]. Research from Palliative Medicine in Oncology also suggests it may improve patient survival [35]. Despite these successes, Palliative Medicine continues to face many challenges. Programs are underdeveloped in low- to middle-income countries, while advanced health care systems struggle to supply a Palliative Medicine workforce to meet the demand for services in urban settings and large academic medical centers.

### 3.1. Impact of Early Involvement of Palliative Medicine

When used early, Palliative Medicine can improve patient quality of life and reduce costs in healthcare. The Center to Advance Palliative Care evaluated trends in Palliative Medicine programs and cost savings through different time periods [24]. Palliative Medicine assists in the reduction of hospital costs by offering customized and intensive services to a small, yet high-cost proportion of patients. Palliative Medicine consultation has reduced hospital costs by $1700 per admission for patients discharged alive and approximately $5000 per admission for patients who died [36]. Additionally, early Palliative Medicine consultation can reduce the cost of hospital stays for patients admitted with advanced cancer diagnoses by up to 24% [37]. Patients with early Palliative Medicine consultation are more likely to receive care outside of the ICU, and are less likely to be hospitalized repeatedly and enroll in Hospice earlier [38].

### 3.2. Impact of Inpatient Palliative Medicine

In the inpatient Palliative Medicine unit, the cost of care can be reduced up to 74% as compared to patients dying outside of the unit [39]. Inpatient Palliative Medicine units have the potential to get busy quickly as demonstrated by Mount Sinai, where 1000 patients were admitted within the first two years of opening with significant cost savings per patient compared to the general hospital wards [40]. The rapid growth of inpatient units and the measurement of their cost savings and outcomes could be further harnessed through standardization of care and patient referral through care pathways.

### 3.3. Impact of Palliative Medicine at End-of-Life

Health care utilization and costs are very high for cancer patients at the end-of-life. In these patients, Palliative Medicine can decrease readmission rates and direct hospital costs by 22–32% within two days of inpatient consults for patients with high co-morbidity scores [37,41]. Cost savings decrease as the duration of time increases from admission to Palliative Medicine consult for patients with advanced cancer [37,42,43]. Institutional annual savings can range in the millions of dollars from a decreased length of stay, reduction in hospital risk-adjusted mortality indices and lowering of daily hospital costs [44,45].

### 3.4. Impact of Outpatient Palliative Medicine

Most of the current data related to cost savings results from inpatient Palliative Medicine consults. The data collected for outpatient Palliative Medicine clinics show a decrease in hospital admission and cost, and improvement in Hospice utilization and patient quality of life within three months of being seen in the clinic [46,47]. Palliative Medicine outpatients visit the emergency room less, have fewer hospitalizations in the last 30 days of life and fewer hospital deaths [48]. Less aggressive care is a described characteristic of Palliative Medicine patients and those followed in clinic generally have longer stays in Hospice [49]. A more recent meta-analysis of eight randomized control trials of outpatient specialty Palliative Medicine clinics found that patients seen in Palliative Medicine cancer care clinics had a 14.1% increase in one-year survival and a higher reported quality of life [50].

### 3.5. Consideration of Caregiver Support Metrics

Most hospitals and healthcare systems measure patient and clinician metrics. However, caregiver and family satisfaction is equally as important to thrive in a competitive healthcare marketplace. Palliative Medicine support of caregivers of patients with advanced cancer demonstrated lower depression screening scores and stress burden when a telemedicine intervention was provided. Care pathways should be considered not only for patient care management but also for interventions with caregivers. Care pathways are also ripe for development for the care of the bereaved [20].

## 4. Conclusions

The field of Palliative Medicine has moved significantly upstream to include the care of any patient with a serious illness. Palliative Medicine and care pathways are impacting professional practice, patient outcomes, quality of care, length of stay and hospital costs. The future of Palliative Medicine should include the development of primary and integrated care pathways for inpatient-, outpatient- and community-based serious illness care, to standardize referral patterns and demonstrate its impact on resource utilization. Just as end-of-life care pathways standardized symptom management at the time of death, similar care pathways should be developed for early Palliative Medicine consultations to address symptom control and cohesive disease management as part of multidisciplinary teams. Care pathway development should also involve support patterns for caregivers and management of the bereaved after hospice enrollment. Outcomes research involving integrated care pathways in Palliative Medicine is completely lacking and should be considered a prime area of future research efforts to support the management of Palliative Medicine programs and further demonstrate the specialty’s positive impacts on patient quality and cost of care.
